# Supplementary material for: Metabolic Profiling and Antioxidant Analysis for the Juvenile Red Fading Leaves of Sweetpotato
Source: Plants (Basel). 2022 Nov 8;11(22):3014. doi: 10.3390/plants11223014 (PMC9697311; doi:10.3390/plants11223014)
Supplement: Supplementary file 1 [file plants-11-03014-s001.zip › sup/Sup Fig of MS-Metabolic profiling 2022-9-23.pdf]

Figure S1.

A

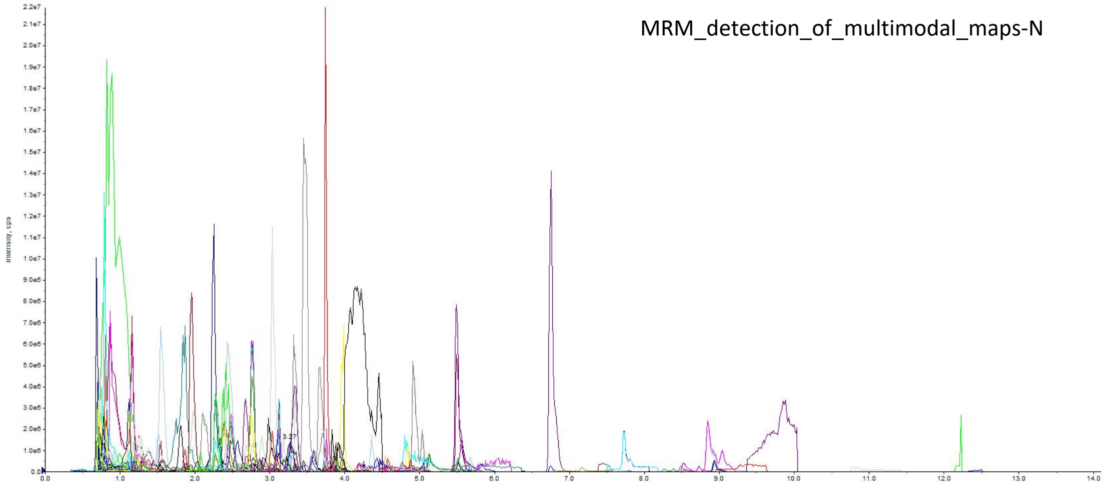

B

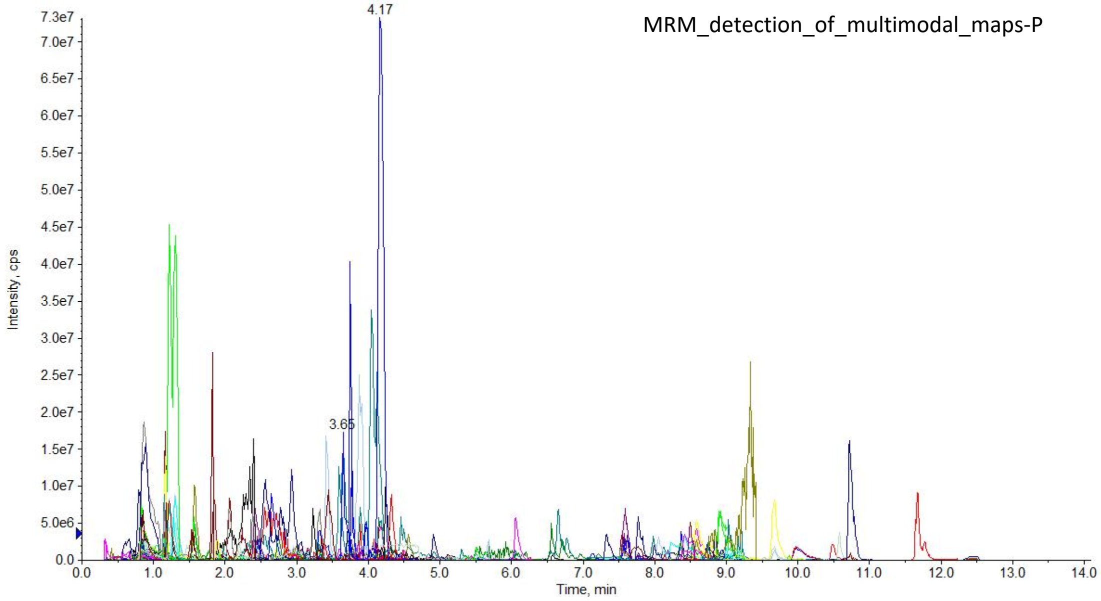

Figure S1 Metabolite detection multipeak map derived from the mode of multiple reaction monitoring (MRM). (A) The detection in negative ion mode and (B) positive ion mode. The X-axes are the retention time (Rt) of the metabolite detection. The Y-axes are the ion current intensity of the ion detection, and the intensity units are counts per second (cps).

Figure S2.

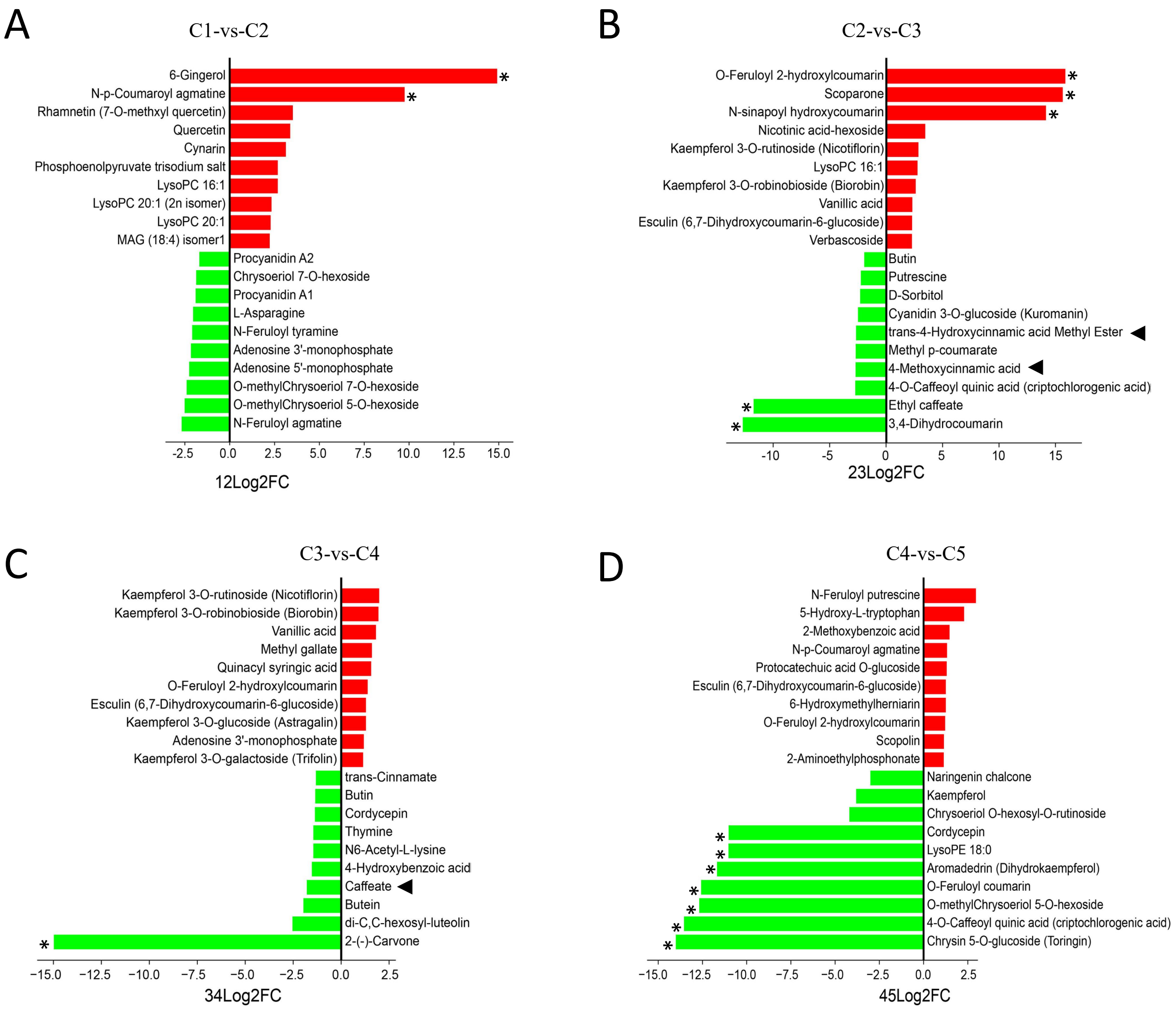

Figure S2 The top 10 differential up- and down-DMs accumulating in adjacent leaf stages.

For C1-vs-C2 (A), C2-vs-C3 (B), C3-vs-C4 (C), and C4-vs-C5 (D) , the red and green columns representing the up-DMs and down-DMs, respectively. The compounds marked with * had |log2FC| greater than eight, and those marked with triangles were among the most abundant 50 metabolites.

Figure S3.

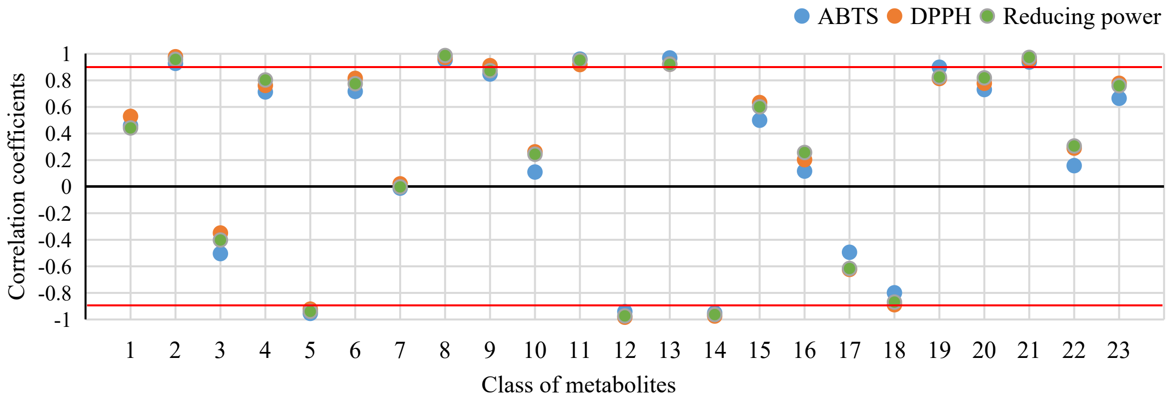

Figure S3 Correlation between metabolite classes and the antioxidant capacities.

The Y-axis shows the Pearson's correlation coefficients (R) of 23 metabolite classes with the antioxidant abilities. The red lines indicate the significance threshold ($R > 0.9$ or $R < -0.9$).
